# Determination Methods of the Risk Factors in Food Based on Nanozymes: A Review

**DOI:** 10.3390/bios13010069

**Published:** 2022-12-31

**Authors:** Yihan Lang, Biao Zhang, Danfeng Cai, Wanjun Tu, Jingyi Zhang, Xuping Shentu, Zihong Ye, Xiaoping Yu

**Affiliations:** Zhejiang Provincial Key Laboratory of Biometrology and Inspection & Quarantine, College of Life Sciences, China Jiliang University, Xueyuan Street, Xiasha Higher Education District, Hangzhou 310018, China

**Keywords:** nanozyme, food hazards, biosensor, food safety, determination

## Abstract

Food safety issues caused by foodborne pathogens, chemical pollutants, and heavy metals have aroused widespread concern because they are closely related to human health. Nanozyme-based biosensors have excellent characteristics such as high sensitivity, selectivity, and cost-effectiveness and have been used to detect the risk factors in foods. In this work, the common detection methods for pathogenic microorganisms, toxins, heavy metals, pesticide residues, veterinary drugs, and illegal additives are firstly reviewed. Then, the principles and applications of immunosensors based on various nanozymes are reviewed and explained. Applying nanozymes to the detection of pathogenic bacteria holds great potential for real-time evaluation and detection protocols for food risk factors.

## 1. Introduction

Agriculture and food production are directly related to the survival and development of mankind. Food safety remains one of the most crucial issues globally because food could be contaminated at all stages of production, packaging, storage, transportation, and value-added processing, giving rise to outbreaks of foodborne diseases [1]. The World Health Organization (WHO) pointed out that an estimated 600 million—almost 1 in 10 people in the world—fall ill after eating contaminated food and 420,000 die every year, resulting in the loss of 33 million healthy life years (DALYs). Food hazards have a variety of factors, such as plant, animal, and microbial metabolites, soil and water pollution hazards from the environment, and purposefully added illegal additives, generated during food packing and processing [2,3]. The review was organized by the category of food risk factors, including pathogenic microorganisms, toxins, heavy metals, pesticide residues, veterinary drugs, and others [4].

At present, the frequently used detection immunoassays, such as high-performance liquid chromatography (HPLC), gas chromatography (GC), mass spectrometry (MS), gas chromatography/mass spectrometry (GC-MS), liquid chromatography/mass spectrometry (LC-MS), and enzyme-linked immunosorbent assay (ELISA), have the advantages of outstanding specificity and accuracy [5]. However, they require tedious pretreatment steps, expensive instruments, specialized technical personnel, and a long testing cycle, which are inappropriate for point-of-care testing (POCT) [6]. In such contexts, with the rapid development and deepening understanding of nanotechnology, nanomaterial-based biosensors to detect food contamination and food adulteration have revolutionized the global food industry [7,8,9].

Nanozymes are artificial nanomaterials with intrinsic enzyme-like properties, which are distinct from “nano-enzymes” with natural enzymes or catalytic ligands immobilized on nanomaterials. On the whole, natural enzymes are readily digested by proteases and lose their enzymatic activity after exposure to extreme pH and high temperatures, which considerably impedes their practical application [10]. Nanozymes are structurally stable and capable of catalyzing reactions not only under mild physiological conditions but also retaining enzymatic activity in extreme environments. For instance, peroxidase substrates could be catalyzed by iron-based nanozymes at extreme pH (1–12) and temperature (−20–80 °C). The term “nano-zyme” was coined by Pasquato et al. in 2004 to employ triazacyclonane-functionalized gold nanoparticles as catalysts for transphosphorylation reactions [11]. In 2007, Yan et al. reported that Fe_3_O_4_ nanoparticles possessed the inherent catalytic activity of horseradish peroxidase (HRP), which could catalyze the conversion of substrates as natural peroxidase under mild physiological conditions [12].

To date, the catalytic performance of nanozymes have been extended from the initial single oxidoreductase (peroxidase) to the current four categories, including oxidoreductase, hydrolase, lyase and isomerase [13,14]. Dozens of inorganic nanomaterials have been found to hold different catalytic activities, for instance, cerium dioxide nanoparticles and ferromagnetic nanoparticles with peroxidase activity, gold nanoparticles with oxidase activity and cadmium sulfide and cadmium selenide nanoparticles with nitrate reductase activity [15,16]. The most outstanding feature of nanoparticles is their superior catalytic activity, low cost, high stability, and controllable and adjustable enzyme activity, which is unmatched by other simulated enzymes [17]. In addition, nanozymes can be size-controlled and surface-modified utilizing sophisticated nanotechnology to modulate their enzymatic activities, which is thought to be an inorganic material with unique physicochemical properties [18]. The discovery of nanozymes breaks the previous notion that inorganic nanomaterials are inert and reveals that they also hold catalytic activities similar to enzymes. Extensive experiments have confirmed that nanozymes are intended to be applied as an alternative to enzymes in the life sciences and the food industry (Table 1) [19,20]. However, the scant biometric events, inadequate water solubility, rational batch design, and catalytic mechanisms of synthetic enzymes based on nanomaterials and the lower catalytic efficiency of some nanozymes compared to natural enzymes are still the prime hindrances confining their applications [21].

At present, sustainably and environmentally conscious lifestyles are gradually being emphasized; therefore, it is particularly crucial to establish a testing system to ensure food safety. Based on the international publications, the main purpose of this paper is to highlight new research data on food hazard detection by nanozymes, summarize the general conclusions, and put forward our views on future development.

## 2. Pathogenic Microorganism

**Figure 1:**
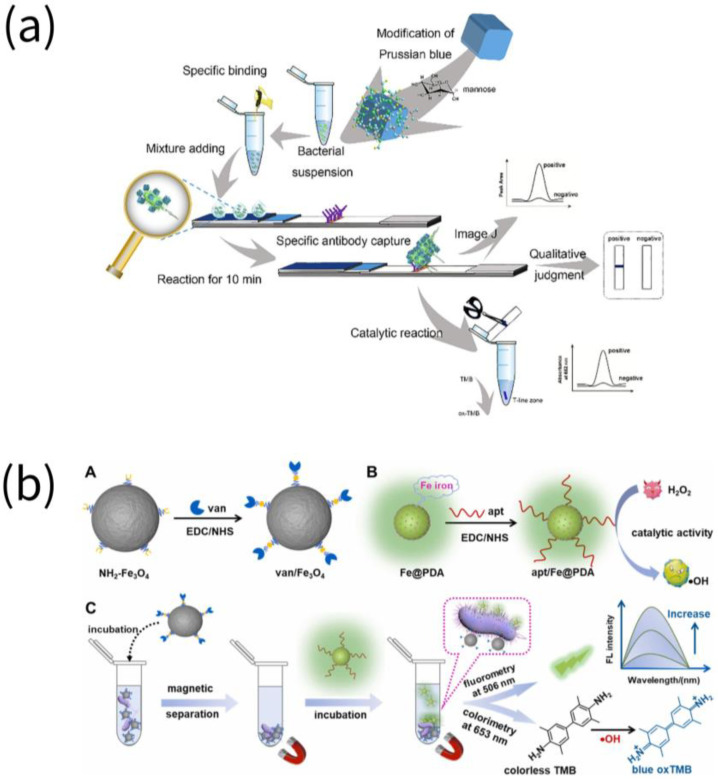
Assays for the detection of pathogenic micro-organisms in food by nanozymes. (**a**) *E. coli* O157:H7 were detected using mannose modified Prussian blue (man-PB) (reprinted with permission from [30], Copyright 2020, Elsevier). (**b**) *Listeria monocytogenes* were detected using Fe@PDA-based nanozyme (reprinted with permission from [32], Copyright 2022, Elsevier).

While physical and chemical contaminations also lead to foodborne diseases, biological contaminants, particularly microorganisms, are considered the greatest danger to food safety [22]. The majority of current conventional procedures for identifying and locating these pathogenic microorganisms are based on colony counting and cell cultures, which requires at least 3–4 days to obtain a presumptive result and about 7 days to produce a definitive identification summary [23]. *Escherichia coli*, *Salmonella enterica*, *Campylobacter jejuni*, *Staphylococcus aureus*, *Listeria monocytogenes* and *Bacillus cereus* are the main bacterium responsible for foodborne illnesses [24]. The search for disposable devices capable of in situ, rapid, and multiplex quantitative detection of pathogenic microorganisms has become a recent research trend, and several rapid determination methods are being more frequently applied, including polymerase chain reaction (PCR) [25]. Due to their speed and reliability, nanomaterials are being extensively utilized as transducing components for biosensors, potentially turning out in situ detection devices for biosecurity as well as clinical and food diagnostics (Figure 1) [26].

A brief description of the single detection mode or single-target nanozyme biosensors is given below. Hu et al. utilized the immune Ps-Pt (IPs-Pt) by growing platinum nanoparticles (Pt) on the surface of carboxyl-functionalized polymer nanospheres (Ps), establishing a quick and accurate colorimetric method for the detection of *Salmonella typhimurium*. The Pt gave the Ps-Pt an ultrahigh peroxidase-mimetic catalytic activity, and the carboxyl group allowed Ps-Pt to bind to streptavidin through covalent binding, with excellent activity and stability [27]. Taking advantage of BSA as a template, Liu et al. published a study of a Co_3_O_4_ magnetic nanozyme (Co_3_O_4_ MNE) with peroxidase-mimetic activity which binds with a unique fusion phage protein. The Co_3_O_4_ MNE@fusion-pVIII particles were able to capture *Staphylococcus aureus* (*S. aureus*) and magnetically separate it from the milk [28]. For the semi-automatic detection of *Salmonella typhimurium*, an enzyme-free optical biosensor based on porous gold@Platinum nanocatalyst (Au@PtNCs) and a passive 3D micro-hybrid was created. It was able to magnetically separate 99% of the target bacteria from the sample in about 10 min. This study used immunomagnetic nanoparticles to separate target Salmonella cells, then immune Au@PtNCs were labeled onto the target cells and catalyzed with H_2_O_2_-3,3′,5,5′-tetramethylbenzidine (TMB) to signal output, and absorbance was measured at 652 nm to calculate the number of bacteria [29].

Multitarget and multimode detection is also one of the popular research directions. For the sake of developing an antibody-free LFIA with three signal readout modes for detecting *E. coli* O157:H7, Wang et al. proposed functional nanozyme/mannose-modified Prussian blue (man-PB) as a novel recognition reagent. The antigenic determinants on the surface of bacteria were more fully exposed as a result of the targeted binding of man-PB to the FimH protein in *E. coli* O157:H7 flagella, which improved the effectiveness of antibodies in recognizing the target bacteria [30]. Utilizing platinum/palladium nanoparticles as signal reporter molecules and a smartphone as a result recorder, Cheng et al. announced a quantitative dual fluorescence immunoassay for the simultaneous detection of two pathogens in milk. The peroxidase-like catalytic activity of Pd @ Pt nanoparticles was utilized for signal enhancement and dual detection in parallel design to eliminate cross-interference, resulting in a significant increase in sensitivity [31]. The signal output of various modes also holds a lot of promise concurrently. Based on Fe-doped polydopamine (Fe@PDA) with significant peroxide-mimetic enzymatic activity and the capacity to emit green fluorescence, a fluorescent/colorimetric dual-mode determination method was developed. The platform contained *Listeria monocytogenes* (*L. monocytogenes*)-recognizing aptamer-modified Fe@PDA (apt/Fe@PDA) and vancomycin-functionalized Fe_3_O_4_ (van/Fe_3_O_4_). Residual *L. monocytogenes* in environmental water were successfully detected with an LOD of 1.0 CFU/mL for fluorescence and 2.3 CFU/mL for colorimetric detection [32].

## 3. Toxins

**Figure 2:**
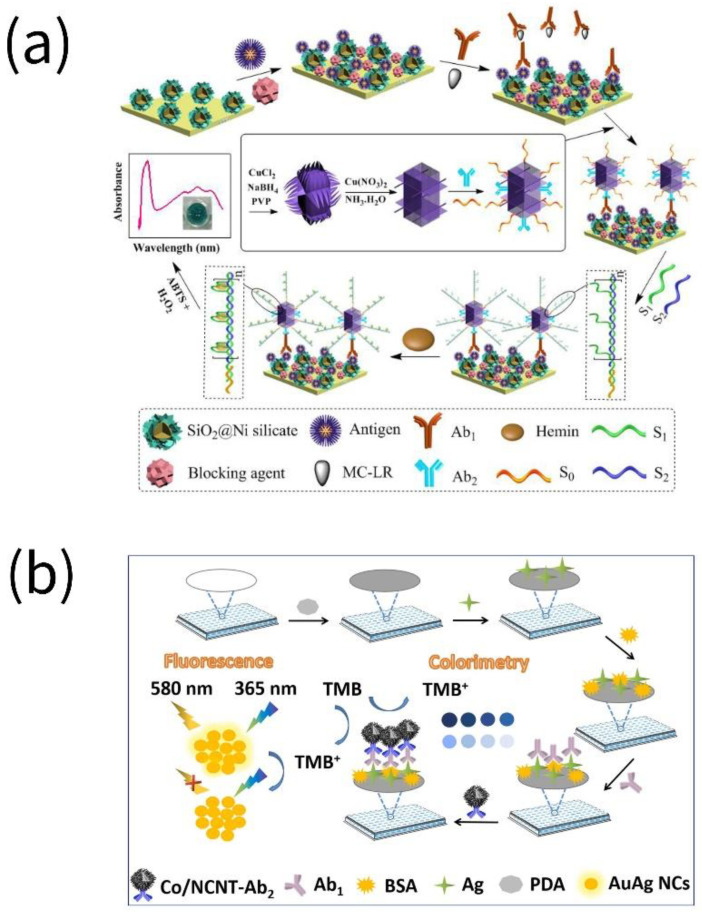
Assays for the detection of toxins in food by nanozymes. (**a**) Microcystin-LR (MC-LR) was detected using coupling copper hydroxide nanozyme and G-quadruplex/hemin DNAzyme (reprinted with permission from [38], Copyright 2019, American Chemical Society). (**b**) OTA was detected using ZIF-derived hollow Co/N-doped carbon nanotubes (CNTs) (reprinted with permission from [40], Copyright 2022, Elsevier).

Toxins generated by pathogens released into food are thought to be the main cause of foodborne disease outbreaks. These toxins induce cytotoxicity by changing the physiological activity and integrity of the plasma membrane [33]. Toxins have a variety of forms depending on where they originate, including bacterial toxins, mycotoxins, phycotoxins, and algal toxins, among which aflatoxin B1 (AFB1) and ochratoxin A (OTA) are the most toxic toxins. The former is one of the culprits of liver cancer and categorized as a Group I carcinogen by the International Agency for Research on Cancer (IARC), while the latter is categorized as a Group 2B carcinogen due to its nephrotoxicity and carcinogenicity [34,35]. Moreover, toxins persist in food even after the relevant pathogens have died, and prompt detection of low amounts of toxins is crucial for reducing poisoning and maintaining public health [36]. Existing toxin detection techniques are sensitive and specific, similar to pathogen detection techniques; however, they require additional instrumentation and operators. Advanced techniques applying nanomaterials as portable biosensors are essential for rapid field detection of specific pollutants (Figure 2).

Making use of MIL-88, with considerable Fe-MOFs-mimetic catalytic activity, and replacing natural enzymes such as HRP to catalyze TMB, an indirect competitive MOF-linked immunosorbent assay (MOFLISA) was developed for the high-throughput and high-sensitivity detection of AFB1. The approach is 20 times more effective than the standard ELISA and could effectively avoid the occurrence of false positives and false negatives during the detection of AFB1 [37]. Liu et al. constructed a microcystin-LR (MC-LR) immunosensor utilizing a double-integrated mimic nanozyme by coupling copper hydroxide nanozyme with G-quadruplex/hemin DNAzyme. Outstanding peroxidase activity was demonstrated by the double-integrated enzyme for the chromogenic reaction of 2,2′-azinobis-(3-ethylbenzothiazoline-6-sulfonic acid) (ABTS) [38].

The multimodal analysis method is more accurate and reliable due to the independence and complementarity of signals. PBNPs produced in situ on the surface of magnetic nanoparticles (MNPs) were taken by Lu et al. to build up a multimodal nanozyme-linked immunosorbent assay (NLISA) that included photothermal, colorimetric, and fluorescence analyses for testing AFB1. The introduced MNPs served as both loading carriers and precursors, quenched the fluorescence of Cy5, and then the generated PBNPs restored the fluorescence of Cy5. The photothermal and colorimetric signals could be applied for POCT and the fluorescence signal for ultrasensitive detection with an LOD of 0.54 fg/mL [39]. Chen et al. prepared Co nanoparticles/N-doped carbon nanotubes (Co/NCNT) with a hollow core/shell structure. While the hollow structure promoted the binding of the active center to the substrate and sped up the reaction, the synergistic effect of Co NPs and NCNT boosted the simulated oxidase activity. Additionally, Co/NCNT produced blue TMB^+^, which quenched the fluorescence of AuAg nanoclusters (NCs) through the internal filtration effect (IFE). A colorimetric-fluorescent dual-mode immunosensor with an LOD of 0.21 ng/L (colorimetric) and 0.17 ng/L (fluorescent) for the sensitive detection of OTA was established [40]. Based on Cu_2_O@Fe(OH)_3_ yolk-shell nanocages with catalytic activity similar to peroxidase, Zhu et al. developed a bimodal multicolorimetric and proportional fluorescence immunosensor capable of sensitively detecting OTA. Cu_2_O@Fe(OH)_3_ efficiently etched Au nanorods (Au NRs), yielding observable color changes and LSPR shifts. It also quenched the emission peak of carbon dots (CDs) at 424 nm and produced a new emission peak at 563 nm to form a ratiometric fluorescence signal [41].

## 4. Pesticide Residues

**Figure 3:**
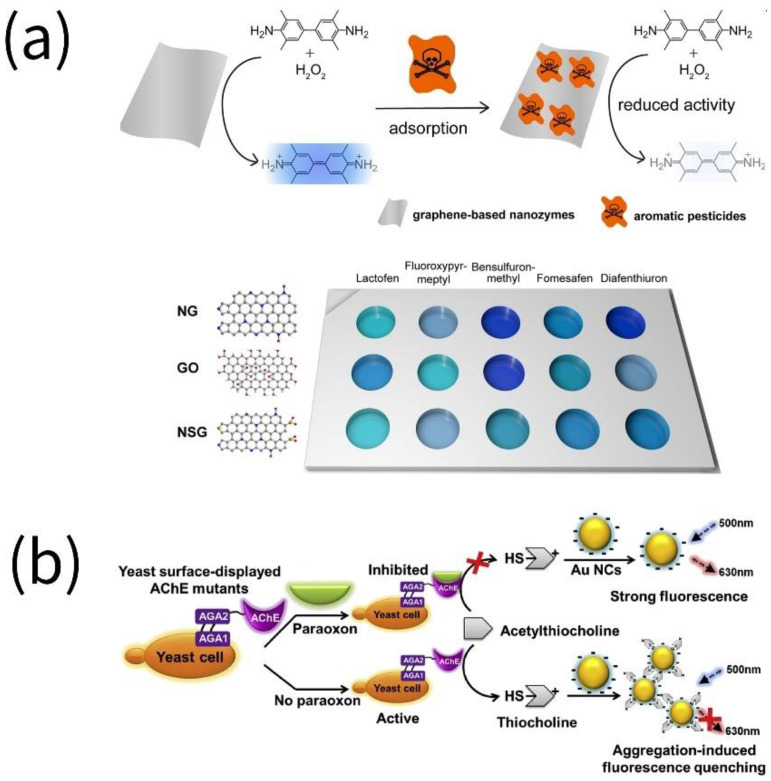
Assays for the detection of pesticide residues in food by nanozymes. (**a**) Five pesticides (i.e., lactofen, fluroxypyr-meptyl, bensulfuron-methyl, fomesafen and diafenthiuron) were detected using heteroatom-doped graphene sensor arrays (reprinted with permission from [51], Copyright 2020, American Chemical Society). (**b**) Paraoxon was detected using fluorescent AuNCs (reprinted with permission from [50], Copyright 2020, Elsevier).

One of the major factors impacting food quality is contamination from pesticide residues [42]. The majority of pesticides are neurotoxic and carcinogenic to individuals, and their residues migrate through the food chain and environment and even persist in nature for more than 15 years [43]. To analyze pesticide residues in agricultural products and the environment at trace or even ultra-trace levels, the sensitive detection of pesticide residues primarily makes use of high-performance liquid chromatography (HPLC), gas chromatography (GC), gas chromatography/mass spectrometry (GC-MS) and HPLC-MS/MS [44]. However, these methods are not appropriate for POCT, while the specific nanozyme structure holds a synergistic effect to raise the sensitivity of detected targets [45] (Figure 3). Pesticides can be broadly divided into four categories based on the chemical property of their active ingredients: organochlorines, organophosphates, carbamates and pyrethroids [46].

Applying a synthetic Fe-N/C SAzyme that directly oxidized TMB to produce blue oxidized product 3,3′,5,5′-tetramethylbenzidine diamine (oxTMB), Ge et al. established a novel, extremely sensitive malathion colorimetric platform. L-ascorbic acid-2-phosphate (AA2P), a substrate of acid phosphatase (ACP), could be hydrolyzed to AA, inhibiting the oxidization reaction of TM and resulting in noticeable blue color fading. With the addition of malathion, AA synthesis was reduced and ACP activity was hampered, restoring the catalytic activity of the single-atom nanozyme [47]. Iron-based metal-organic gel (MOGs) nanosheet hybrids with AuNPs immobilization (AuNPs/MOGs (Fe)) were fabricated to detect organophosphorus (OPs), which displayed excellent chemiluminescence (CL) properties. The considerable enhancement of CL was blamed for the modification of AuNPs on the MOGs (Fe) nanosheet, which synergistically increased the CL reaction by speeding the formation of OH^•^, O_2_^•−^ and ^1^O_2_ [48].

The removal of peroxidase-like activity and color interference is crucial for colorimetric analysis of the nanozyme. The GeO_2_ nanozyme was found that it only possessed peroxidase-like activity but no oxidase-like capability, which rendered the related detection system free from O_2_ disturbance. In addition, the white GeO_2_ nanozyme removed its color interference. Accordingly, a colorimetric sensing platform for ultra-trace detection of OPs pesticides with paraoxon as a representative model was proposed. In the absence of paraoxon, the active acetylcholinesterase (AChE) degraded the GeO_2_ nanozyme and lost its peroxidase function by hydrolyzing acetylthiocholine (ATCh) to thiocholine (TCh). In the presence of paraoxon, AChE was irreversibly inactivated and TCh production was inhibited [49].

While most investigations have been devoted to further optimizing fluorescent probes or assays to enhance the sensitivity for OPs, Liang et al. conducted groundbreaking work on creating yeast-surface-displayed acetylcholinesterase (AChE) mutants (E69Y and E69Y/F330L) from the perspective of modifying the sensitivity of AChE for OPs. Using electronegative fluorescent gold nanoclusters (AuNCs) combined with AChE mutants, an ultra-trace fluorescence assay for OPs with the LOD of 3.3 × 10^−14^ M was established, indicating that the E69Y and F330L mutations had the potential to significantly improve the sensitivity of the nanozyme to OPs [50].

## 5. Veterinary Drugs

**Figure 4:**
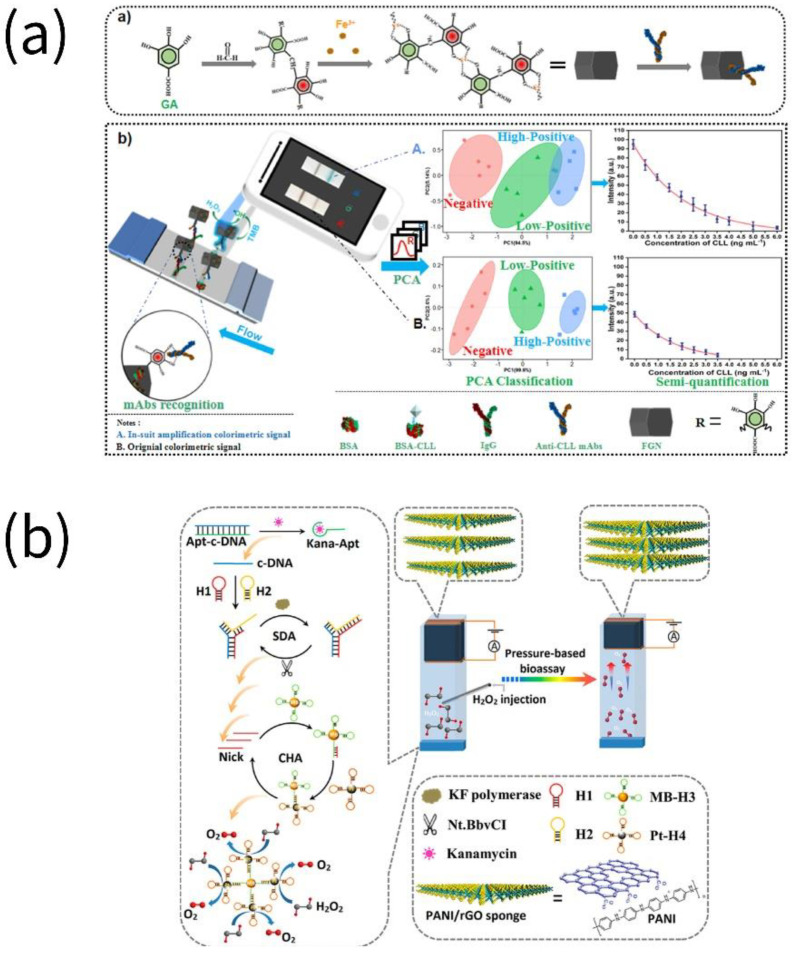
Assays for the detection of veterinary drugs in food by nanozymes. (**a**) Clenbuterol was detected using Fe-GA nanozymes (reprinted with permission from [55], Copyright 2022, Elsevier). (**b**) Kanamycin was detected using polyaniline-nanowire-functionalized reduced-graphene-oxide (PANI/rGO) framework (reprinted with permission from [57], Copyright 2018, American Chemical Society).

Veterinary drugs are the substances applied to prevent, treat and diagnose animal diseases or purposefully regulate animal physiological functions and effectively help farmers solve the problems of livestock threatened by epidemics in breeding production, including antibiotics, antiparasitic and antifungal drugs, hormones, growth promoters, etc. [52]. In the ongoing expansion of the livestock industry today, the employment of veterinary drugs has been crucial in the production of animal-derived food [53]. Veterinary drug residues are the substances of prototype drugs and their metabolites as well as related impurities that accumulate or remain in the organisms or products of livestock and poultry (such as eggs, milk, meat, etc.) following drug application (Figure 4) [54].

Li et al. established the Fe-gallic acid (GA) nanozymes (FGN), an artificial multi-iron peroxidase with monoclonal antibody recognition activity and high catalytic performance inspired by polyphenol–protein interactions. Afterward, clenbuterol (CLL) in pork and poultry was determined applying the nanozyme-mediated dual colorimetric immunochromatographic in conjunction with smartphones, with a detection limit of 0.172 ng mL^−1^ [55]. Applying aggregation-induced (AI)-electrochemiluminescence (ECL)-containing covalent organic framework materials (COF-AI-ECL) as the signal element and Co_3_O_4_ nanozyme as the signal amplification component, a CAP molecularly imprinted sensor was established. Co_3_O_4_ catalytically amplified the ECL signal of COF-AI-ECL, which was effectively quenched by CAP; thus, the ECL signal was controlled by the elution and adsorption of CAP by molecularly imprinted polymer (MIP) [56]. Kanamycin (Kana) was detected on polyaniline-nanowire-functionalized reduced-graphene-oxide (PANI/rGO) framework by catalyzing H_2_O_2_ to generate oxygen using platinum nanozymes on hairpin DNA probes. The principle of signal amplification was to produce a sizable amount of Pt nanoparticles through the coupling of catalytic hairpin assembly (CHA) reaction and strand-displacement amplification (SDA) reaction [57].

Multisignal, enhanced ultrasensitive detection of veterinary drugs is considered an advanced academic research achievement. Utilizing planar VS_2_/AuNPs nanocomposites as the electrode sensing platform, streptavidin-functionalized CoFe_2_O_4_ nanozyme, and methylene-blue-labeled hairpin DNA (MB-hDNA) as signal-amplifying components, an electrochemical aptamer sensor for kanamycin (Kana) proportional detection was developed. The VS_2_/AuNPs nanocomposites were combined with hDNA complementarily hybridized with biotinylated Kana-aptamers, and the CoFe_2_O_4_ nanozyme immobilized on the aptamer sensor possessed excellent peroxidase-like catalytic activity. In the presence of Kana, aptamer biorecognition resulted in a decrease in nanozyme accumulation and an increase in the response of MB [58].

## 6. Heavy Metals

**Figure 5:**
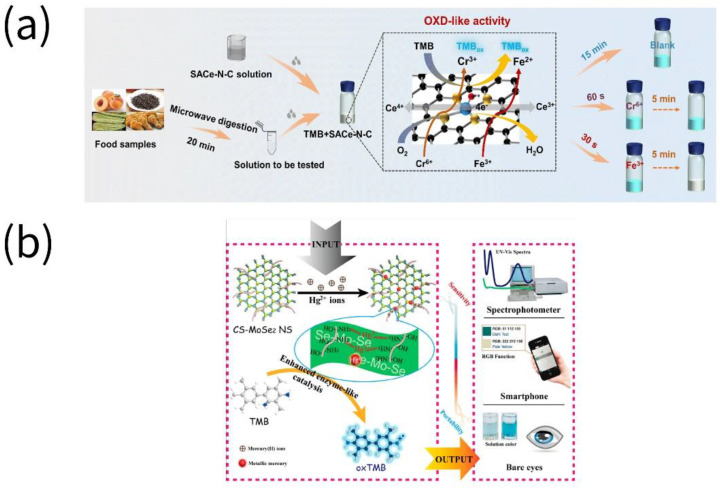
Assays for the detection of heavy metals in food by nanozymes. (**a**) Fe^3+^ and Cr^6+^ were detected using single-atom Ce-N-C (SACe-N-C) (reprinted with permission from [67], Copyright 2022, Elsevier). (**b**) Hg^2+^ was detected using chitosan-functionalized molybdenum (IV) selenide nanosheets (CS-MoSe_2_ NS) (reprinted with permission from [68], Copyright 2019, American Chemical Society).

Excessive levels of heavy metal content in food are caused by heavy metal enrichment in the food chain due to sewage irrigation, automobile exhaust emissions, and the massive application of fertilizers and pesticides [59]. The main hazardous heavy metal ions (HMIs) include Ni^2+^, Cr^6+^, Pb^2+^, Hg^2+^, Cd^2+^ and Cu^2+^, and long-term exposure to these HMIs up to a certain threshold is associated with several health risks, such as Alzheimer’s disease and metabolic disorders [60]. They also impact the cardiovascular, neurological, and renal systems and cause organic injury to human organs. The traditional methods for detecting heavy metals in food mainly involve atomic absorption spectrometry (AAS), atomic fluorescence spectrometry (AFS), inductively coupled plasma/mass spectrometry (ICP-MS), inductively coupled plasma/atomic emission spectrometry (ICP-AES), and ultraviolet-visible spectrometry (UV-vis Spectrometry) [61,62]. Here are several instances of HMIs detection utilizing nanozyme sensors (Figure 5).

After realizing the remarkable selectivity and high-speed absorption of Hg by CuS nanostructures, Fang et al. first put forward an affordable Hg^2+^ nanosensor. CuS hollow nanospheres (HNSs) were the central component and performed three functions: they acted as a concentration carrier for Hg^2+^ pre-enrichment, a recognition unit for Hg^2+^ sensing, and an amplifier and readout for peroxidase-mimetic signals [63]. Anchoring single-atom Fe on a monolayer of two-dimensional nitrogen-doped graphene, the synthesized SA-Fe/NG held excellent peroxidase-like activity, 100% Fe atom utilization and Fe-N-C structure. Based on the preferential specific interaction of TMB oxidation inhibitor 8-hydroxyquinoline (8-HQ) with Cr(VI), which restored oxTMB to blue color TMB, Mao et al. established a single-atom nanozyme colorimetric sensing method for Cr(VI) [64]. Bipyridine-containing COF nanosheets (Tp-Bpy NSs) with regular pore structure, abundant nitrogen-containing functional groups, and flexible topological connectivity were synthesized. AuNPs were generated in situ to form AuNPs@TP-Bpy. The majority of the anchored AuNPs were exposed to the two-dimensional pore structure of Tp-Bpy NSs, increasing the contact area. It was revealed that the ultra-sensitive detection performance of AuNPs@Tp-Bpy nanocomposites for Hg^2+^ was attributed to the synergistic effect of the enhanced catalytic activity of gold amalgam and the considerable access probability of TP-Bpy nanosheets for Hg^2+^ [65].

Regarding sensitive and rapid POCT for a wide range of HDMIs in food and environment, there are still a lot of technical barriers that need to be solved. Applying porous Co_3_O_4_ nanodisks with fairly strong peroxidase-like activity to detect multiple heavy metals, a colorimetric sensor was formed. The electron transferred between the porous Co_3_O_4_ nanodisks and TMB was blocked and the catalytic activity was significantly inhibited by HDMIs (Cd(II), Hg(II), Pb(II) and As), indicating that heavy metals had anti-competitive inhibition of peroxidase activity. The LOD values of this assay were 0.085 μg L^−1^ for Cd(II), 0.19 μg L^−1^ for Hg(II), 0.22 μg L^−1^ for Pb(II) and 0.156 μg L^−1^ for As. It is worth noting that when the concentration of heavy metals exceeded 5 μg L^−1^ or the change of absorbance was greater than 0.5, it could be distinguished by the naked eye [66].

## 7. Others

**Figure 6:**
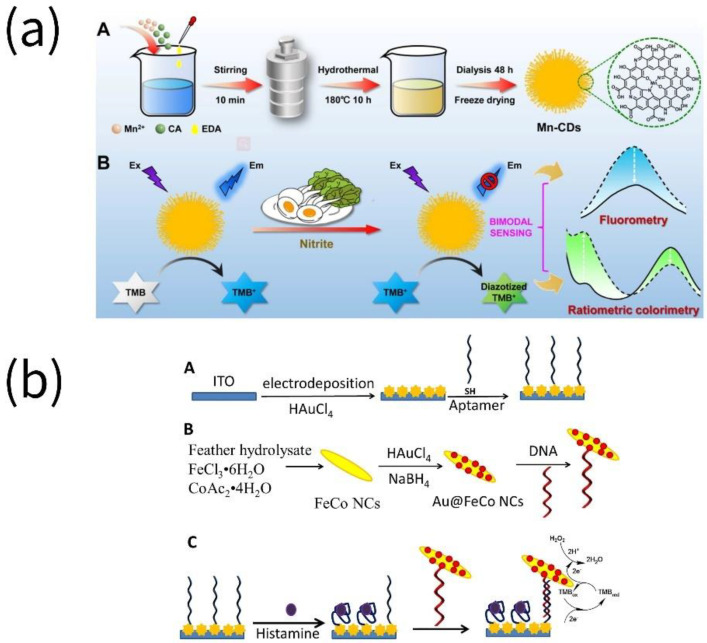
Assays for the detection of other food hazard substances in food by nanozymes. (**a**) Nitrite was detected using Mn-doped N-rich carbon dots (Mn-CDs) (reprinted with permission from [78], Copyright 2022, American Chemical Society). (**b**) Histamine was detected using DNA/Au@FeCo nanoflowers (NCs) (reprinted with permission from [74], Copyright 2022, Elsevier).

Along with the aforementioned common food hazards, there are allergens, food additives, and illegal food additives that should not be ingested in excess and are damaging to the human organism [69]. Novel nanozyme biosensors for sensitive detection of histamine (HA), nitrite and Sudan are given below (Figure 6).

HA is a substance produced by the decarboxylation of histidine, a biogenic amine generated by the spoilage of high-protein foods, and plays an important role in inflammation and allergic reactions in humans [70]. Fish products are an integral component of the human diet, but fish is highly perishable, and histidine can be readily converted to HA through decarboxylation if transported or stored under improper conditions [71]. HA is highly toxic and causes headaches, nausea, and other adverse reactions when ingested in excess, even threatening life in severe cases [72]. Binding PBNPs to goat anti-mouse antibodies for the purpose of forming nanozyme–antibody conjugates, Liao et al. established a nanozyme-mediated ratiometric fluorescence immunoassay for testing HA. The oxidation of o-phenylenediamine (OPD) produced fluorescent 2,3-diaminophenothiazine (oxOPD) under the catalysis of PBNPs, which formed a new emission peak at 570 nm and quenched the fluorescence of CDs at 450 nm [73]. Covalently coupling DNA aptamers with excellent selectivity for HA to a gold nanoflower-modified ITO electrode (apt/AuNFs/ITO) and employing DNA/Au@FeCo NCs with peroxidase-like activity as electrochemical probes, an electrochemical sensor for the determination of HA was developed [74]. Wang et al. published a biomimetic enzyme-linked immunoassay (BELISA) based on the labeled Au@Pt@Au complex nanozyme with peroxidase-mimetic activity for detecting HA [75].

Nitrite exists in a variety of foods, and there are two main sources. First, when vegetables are pickled or cooked, the nitrate in them is reduced to nitrite under the action of reducing bacteria. Second, nitrite has antibacterial and antioxidant properties and thus is widely employed as a food additive in meat and dairy products [76]. However, excessive intake of nitrite has indirect carcinogenic, teratogenic, mutagenic, and other risks to humans, and a single intake of 0.3 g or more causes poisoning or even death [77]. Synthesizing bifunctional Mn-doped N-rich CDs (Mn-CDs) with controlled photoluminescence, high oxidase-like activity, abundant functional groups and favorable hydrophilicity which effectively catalyzed the oxidation of colorless TMB to blue TMB^+^, a bimodal colorimetric/fluorescence technique for the reliable and efficient determination of nitrite in complex matrices was established [78]. Another paper by the same authors investigated the carbon-supported Mn_3_O_4_ particles, utilizing the coupling of a specific diazotization reaction with oxidase-mimetic catalysis, a novel dual-mode and dual-scale electrochemical nitrite sensing method was built up [79]. Adegoke et al. synthesized a chemically modified AuNP-CeO_2_ NP-anchored GO hybrid nanozyme (AuNP-CeO_2_ NP@GO) applied to the catalytic colorimetric detection of nitrite, where AuNP-CeO2 NP@GO yielded high peroxidase catalysis [80].

Sudan is frequently employed as a synthetic pigment in food additives, and the IARC has classified Sudan I, II, III and IV as animal carcinogens [81]. Sudan’s consumption for improving the appearance and color of food products has been outlawed in China by the Food and Drug Administration [82]. A screen-printed electrochemical sensor based on La^3+^-doped Co_3_O_4_ nanocubes was established for the detection of Sudan I, utilizing the excellent catalytic properties, favorable adaptability in catalytic reactions, and interaction with oxygen groups in Co_3_O_4_ of nanozyme [83]. Ye et al. applied CuO nanoparticle-decorated 3D N-doped porous carbon (CuO/3D NPC)-modified electrodes with an excellent electrocatalytic activity of CuO, which exhibited high sensitivity and broad linear range for the detection of Sudan I [84]. A polystyrene-coated, magnetic Fe_3_O_4_ nanoparticle (PSt@Fe_3_O_4_) was employed as a solid-phase adsorbent for liquid–solid extraction prior to magnetic solid phase extraction (MSPE), making it applicable to solid food matrices. The non-polar Sudan dyes were adsorbed onto similarly structured non-polar polystyrene wrapped around nanoparticles and then released Sudan dyes using a desorbent for detection and quantification [85].

## 8. Conclusions and Perspectives

Public health risks related to food safety remain a top priority, and specific instances of nanozyme biosensors for detecting primary food pollutants were discussed. Although some of the drawbacks of conventional detection techniques have been overcome through the development of various nanomaterials, most nanozyme-based sensors require additional manipulation steps that add operational complexity compared to traditional methods, and considerable work remains to be completed before they could become viable alternatives to conventional assays, for instance, innovating the structure of immunosensors, exploring new nanozyme-based sensors, and optimizing existing nanozyme by combining experimental techniques and theoretical strategies. Future nanozyme developments will put more emphasis on being more portable, wide-ranging, and industrialized, such as by combining artificial intelligence (AI) and machine learning with food quality maintenance to facilitate rapid identification of food risk factors and through the proliferation of nanosensors associated with portable mobile devices to enable precise monitoring at home. We hope that this paper could provide new inspiration for the future outlook of nanozyme-based sensors for food safety analysis.

**Table 1:** Nanozyme-based biosensors for the detection of food contaminants.

Analytes	Biosensors	Nanozymes	LODs	Food matrix	Ref.
**Pathogenic microorganism**					
*Escherichia coli O157:H7* (*E. coli* O157:H7)	Colorimetric	Platinum-coated magnetic nanoparticle clusters (Pt/MNCs)	10 CFU/mL	milk	[86]
*E. coli* O157:H7	Colorimetric	Hemin-concanavalin A hybrid nanoflowers (HCH nanoflowers)	4.1 CFU/mL	milk	[87]
*Salmonella Enteritidis*	Colorimetric	Fe-MOF nanoparticles	34 CFU/mL	milk	[88]
*Salmonella typhimurium*	Colorimetric	Prussian blue nanoparticles (PBNPs)	6 × 10^3^ CFU/mL	powdered milk	[89]
*Salmonella enterica serovar typhimurium*	Colorimetric	ZnFe_2_O_4_-reduced graphene oxide nanostructures	11 CFU/mL	milk	[90]
*Listeria monocytogenes (L. monocytogenes)*	Colorimetric	AgNCs	10 CFU/mL	pork	[91]
**Toxins**					
Aflatoxin B1 (AFB1)	Colorimetric	Mesoporous SiO_2_/Au-Pt (m-SAP)	0.005 ng/mL	peanut	[92]
AFB1	Colorimetric	Porphyrin NanoMOFs (NanoPCN-223(Fe))	0.003 ng/mL	milk	[93]
AFB1 and *Salmonella Enteritidis*	Colorimetric/Fluorescent	Pt@PCN-224-HRP-initiator DNA (PP-HRP-iDNA)	6.5 × 10^−4^ ng/mL and 4 CFU/mL for AFB1 and *Salmonella* Enteritidis respectively	rice and milk	[94]
Ochratoxin A (OTA)	Colorimetric	Co(OH)_2_ nanocages	2.6 × 10^−4^ ng/mL	corn	[95]
Saxitoxin (STX)	Colorimetric	AuNPs	4.246 × 10^−4^ nM	shellfish	[96]
Pesticide residues					
Diazinon	Fluorescent	Fe_3_O_4_ nanoparticles@ZIF-8 (Fe_3_O_4_ NPs@ZIF-8)	0.2 nM	water and fruit juices	[97]
Acetamiprid	Colorimetric	Gold nanoparticles (GNPs)	0.1 ng/mL	-	[98]
Methyl-paraoxon	Colorimetric/Fluorescent	nanoceria	420 nM	Semen nelumbinis, Semen Armeniacae Amarum, Rhizoma Dioscoreae	[99]
Paraoxon	Fluorescent	Carbon quantum dots (CQDs)	0.05 ng/mL	tap and river water	[100]
Paraoxon	Fluorescent	MnO_2_ Nanosheet-Carbon Dots	0.015 ng/mL	tap water, river water, rice, and cabbage	[101]
Paraoxon, Parathion, Fenitrothion and Diazinon	Colorimetric	AuNPs	0.13 ng/mL, 0.37 ng/mL, 0.42 ng/mL and 0.20 ng/mL for paraoxon, parathion, fenitrothion and diazinon, respectively	water	[102]
Glyphosate	Colorimetric/Fluorescence/Photothermal	N-CDs/FMOF-Zr	13.1 ng/mL, 1.5 ng/mL and 11.5 ng/mL for colorimetric, fluorescence and photothermal respectively	rice, millet, and soybeans	[103]
**Veterinary drugs**					
Tetracycline (TC)	Colorimetric	AuNCs	46 nM	drugs and milk	[104]
Kanamycin	Colorimetric	Gold nanoparticles (GNPs)	1.49 nM	-	[105]
Enrofloxacin	Chemiluminescence	Co(OH)_2_ nanosheets	4.1 × 10^−5^ ng/mL	shrimp, chicken, and duck meat	[106]
Norfloxacin (NOR)	Colorimetric	1-methyl-D-tryptophan-capped gold nanoclusters (1-Me-D-Trp@AuNCs)	200 nM	drugs	[107]
Sulfaquinoxaline (SQX)	Chemiluminescence	Cu(II)-anchored unzipped covalent triazine framework (UnZ-CCTF)	7.6 × 10^−4^ nM	milk	[108]
Chloramphenicol (CAP)	Electrochemiluminescence	Ultrathin PtNi	2.6 × 10^−7^ nM	pig urine, river water, and milk	[109]
**Heavy metals**					
Hg^2+^	Colorimetric	Pt NP	16.9 nM, 26 nM and 47.3 nM for MilliQ water, tap water and ground waters, respectively	MilliQ water, tap water, and ground waters	[110]
Hg^2+^ and MeHg	Fluorescent	Copper oxide-based nanocomposites	3.0 nM and 3.3 nM for Hg^2+^ and MeHg, respectively	tap water, river water, seawater, and dogfish muscle	[111]
Ag^2+^	Colorimetric	Chitosan-PtNPs (Ch-PtNPs)	4 nM	tap and lake water	[112]
Ag^2+^	Colorimetric	Pt nanoparticles	7.8 × 10^−3^ nM	river water	[113]
Pb^2+^	Colorimetric	Tungsten disulfide (WS_2_) nanosheets	4 ng/mL	tap water, soil, wheat, and fish serum	[114]
Pb^2+^	Colorimetric	Au@Pt nanoparticles	3.0 nM	lake water	[115]
Pb^2+^ and Hg^2+^	Fluorescent	Metal-deposited bismuth oxyiodide (BiOI) nanonetworks	nanomolar quantities	tap water, river water, lake water, and sea water	[116]
**Others**					
Sulfide	Colorimetric	GMP-Cu nanozyme with laccase activity	670 nM	baking soda, rock sugar, konjac flour, and xylitol	[117]
Nitrite	Colorimetric/Electrochemical	Histidine(His)@AuNCs/rGO	2 nM and 700 nM for Colorimetric and Electrochemical respectively	sausage	[118]
Nitrite	Colorimetric	Hollow MnFeO particles	200 nM	sausage, pickles, and salted eggs	[119]
Salbutamol	Colorimetric	AgNPs	2.614 × 10^−4^ ng/mL	tap water and artificial urine	[120]

## Data Availability

The datasets generated for this study are available on request to the corresponding author.
